# Cognitive function, physical function, and mental health in older adults amid reduced primary and specialist healthcare service use during COVID-19: the HUNT study

**DOI:** 10.1007/s11357-025-01909-x

**Published:** 2025-09-27

**Authors:** Tanja Louise Ibsen, Ekaterina Zotcheva, Sverre Bergh, Debby Gerritsen, Gill Livingston, Hilde Lurås, Svenn-Erik Mamelund, Anne Marie Mork Rokstad, Bjørn Heine Strand, Richard C. Oude Voshaar, Geir Selbæk

**Affiliations:** 1 The Norwegian National Centre for Ageing and Health (Ageing and Health), Vestfold Hospital Trust, Postbox 2136, N- 3103 Tønsberg, Norway; 2 Research Centre for Age-related Functional Decline and Disease (AFS), Innlandet Hospital Trust, Ottestad, Norway; 3 Department of Primary and Community Care, Research Institute for Medical Innovation, Radboudum Alzheimer Center, Radboud University Nijmegen Medical Center, Nijmegen, The Netherlands; 4 Division of Psychiatry, University College London, London, UK; 5 Camden and Islington NHS Foundation Trust, London, UK; 6 Health Services Research Unit, Akershus University Hospital, Oslo, Norway; 7 Institute of Clinical Medicine, University of Oslo, Oslo, Norway; 8 Centre for Research On Pandemics & Society (PANSOC), OsloMet - Oslo Metropolitan University, Oslo, Norway; 9 Faculty of Health Sciences and Social Care, Molde University College, Molde, Norway; 10 Department of Physical Health and Ageing, Norwegian Institute of Public Health, Oslo, Norway; 11 Department of Geriatric Medicine, Oslo University Hospital, Oslo, Norway; 12 University of Groningen, Groningen, The Netherlands; 13 Department of Psychiatry, University Medical Center Groningen, Groningen, The Netherlands; 14 Institute of Clinical Medicine, Faculty of Medicine, University of Oslo, Oslo, Norway

**Keywords:** COVID-19, Healthcare services, Cognitive function, Physical function, Mental health, Longitudinal cohort study

## Abstract

COVID-19 containment measures reduced older adults’ healthcare access, with uncertain long-term effects on cognitive, physical, and mental health. To investigate whether reductions in primary and specialist healthcare service use during the pandemic were associated with changes in cognitive, physical, and mental health in community-dwelling older adults, with attention to sex differences. Data from the Norwegian Trøndelag Health Study collected before (HUNT4 70 + , 2017–2019) and after the pandemic (HUNT AiT, 2021–2023) included 5387 participants (53% women) aged 70 + . Propensity score matching, accounting for baseline confounders, was used to examine associations between reduced healthcare service use and cognitive, physical, and mental health changes from pre- to post-pandemic. Reduced contact with general practitioners was associated with greater cognitive decline among women (MoCa-change − 0.32 [95% CI − 0.62, − 0.32]). No differences were observed in physical or mental health. Reductions in other primary care services (e.g., in-home nursing, practical assistance) were associated with greater decline in cognitive function (MoCa-change − 0.94 [− 1.53, − 0.36]), particularly among men (MoCa-change − 2.12 [− 3.13, − 1.11]). Men also had a decline in physical function (SPPB-change − 1.06 [− 1.79, − 0.33]). No differences in mental health were observed. Reductions in specialist healthcare services were unrelated to health changes in the overall sample but linked to improved physical function in women (SPPB-change 0.32 [0.11, 0.53]). Although associations between reduced healthcare service use during the pandemic and cognitive, physical, and mental health were limited, findings highlight the importance of sustaining access to primary care for older adults during public health crises.

**Trial registration** The study is pre-registered at ClinicalTrials.gov 02.02.2021, with the identification number NCT 04792086.

**Supplementary Information:**

The online version contains supplementary material available at 10.1007/s11357-025-01909-x.

## Introduction

The containment measures implemented during the COVID-19 pandemic led to a reduction in the use of healthcare services among older adults around the world [1–5]. A global WHO survey confirmed widespread disruptions in essential healthcare services across nearly all countries [6], and a systematic review of 81 studies reported a median 37% reduction in healthcare utilization during the early pandemic phase [7]. Our recent research on healthcare service use in Norway, however, found that most older adults had increased contact with general practitioners (GPs) during the pandemic. Yet, it also revealed a decline in the use of other primary health care services, such as in-home nursing and day care services, as well as specialist healthcare services, during the first six months following the COVID-19 lockdown, March 12, 2020 [2]. In Norway, the first lockdown lasted from March 12 to June 15, 2020, with strict infection control measures leading to reduced or closed health and care services, transfer of health professionals to COVID-19-related tasks, hospital wards reserved for infected patients, and the use of digital solutions where possible. Services gradually reopened after mid-June [8]. Within other primary care services, the reduction was mainly aimed at minimizing contact between people to limit the spread of the virus. The reduction of specialist healthcare services, such as contact with general hospitals, on the other hand, was intended to free up resources for the pandemic response to care for those infected with COVID-19 [8].

A common concern about the pandemic has been the long-term negative impact of infection control measures, such as reduced healthcare services for older adults. A large body of literature has documented mental health challenges, cognitive decline, and reduced physical function during the pandemic [9–12]. However, most studies on changes in access to healthcare services have been conducted during the first stage of the pandemic [1, 4]. Our recent research, following Norwegian older adults for one and a half years after the lockdown, found that service use returned to pre-pandemic levels one year after the lockdown [2]. Nevertheless, the same population had an increase in medication prescriptions of analgesics and psychotropics two years after the pandemic, indicating an increased need for medical treatment when non-pharmacological interventions were reduced [13]. Thus, we share concerns about the long-term health consequences for those who experienced a reduction in their primary and specialist healthcare services.

A substantial body of literature indicates sex differences in health outcomes [14–18] and health care utilization [19, 20] in older adults. Studies have shown that women experience a steeper cognitive decline than men [14, 17, 18], face greater disadvantages in physical functioning [16] and are more likely to report mental health symptoms such as depression and anxiety [15]. Additionally, due to higher rates of multimorbidity, women tend to use health care services more frequently than men [19, 20]. This knowledge highlights the importance of considering sex differences when investigating health outcomes related to changes in health care service use.

In this prospective, population-based study, we aimed to investigate how the reduction in primary and specialist healthcare services during the pandemic was associated with the cognitive function, mental health, and physical function of older adults aged 70 years and above, using propensity score matching. Additionally, we explored whether these relationships varied between men and women.

## Methods

### Study design

Our study utilized data from a longitudinal, population-based cohort of individuals aged 70 years and older, derived from the Norwegian Trøndelag Health Study (HUNT4 70 +), conducted between 2017 and 2019, and its follow-up, the HUNT Ageing in Trøndelag study (HUNT AiT), conducted between 2021 and 2023. HUNT is a large-scale, population-based health survey that has invited all adult residents of the former Nord-Trøndelag County to participate across four waves. Participants completed questionnaires covering socio-demographic and clinical information and healthcare professionals conducted comprehensive clinical assessments conducted by healthcare professionals [21]. In the present study, participant data was linked to information on healthcare utilization from two national registries. First, the Norwegian Registry of Primary Health Care (KPR) [22], and second, the Norwegian Patient Registry (NPR) [23].

### Participants

The HUNT4 70 + cohort included 9,930 individuals, of whom 5708 later took part in HUNT AiT. Individuals participating in both HUNT4 70 + and HUNT AiT formed the basis of our study sample. From this group, we excluded the 197 nursing home residents and 124 individuals with missing data on key study variables in one of the two study periods. This resulted in a final study sample of 5387 individuals.

### Data collection

We used individual data on age (birthyear), sex (women vs. men), education (primary, secondary, and tertiary) cohabitation status (living alone vs. living together with someone), and comorbidity (0–1 self-reported disease vs. 2 + self-reported diseases [24]) collected at baseline at HUNT4 70 +. Cognitive function, physical function, and mental health data were collected at baseline and follow-up (HUNT AiT). Data from the two registries, KPR and NPR, were linked to participants’ data using the Norwegian personal identification number. Data on primary health care services included contact with GPs, in-home nursing, practical assistance, daycare services, respite care, short-term nursing home stays, municipal housing, and nursing home admissions. In this paper, we have divided primary healthcare services into contact with GPs and other primary care services. Data on specialist health care services included contact with general hospital services. In this study, we utilized data on healthcare service use from March 12, 2019, until March 11, 2021.

### Outcome measures

Cognitive function was assessed using the Montreal Cognitive Assessment (MoCA), a comprehensive 30-point tool designed to evaluate cognitive status across six key domains: visuospatial construction, executive function, episodic memory, attention, language, and orientation. Higher scores indicate better cognitive function [25]. We created a variable measuring the change in cognitive function by subtracting the MoCA score at baseline from the MoCA score at follow-up.

Physical function was assessed using the Short Physical Performance Battery (SPPB). The SPPB is based on three timed tasks: standing balance, walking speed, and chair stand tests. The score ranges from 0 to 12 points, and a higher score indicates better physical function [26]. We created a variable measuring the change in physical function by subtracting the SPPB score at baseline from the SPPB score at follow-up.

Mental health was assessed using the Cohort of Norway Mental Health Index (CONOR-MHI), which includes seven items rated on a 1–4-point scale (total score range: 7–28). The items cover being nervous and unsettled, troubled by anxiety, secure and calm, irritable, happy and optimistic, sad/depressed, and lonely during the last two weeks. The average score across all seven items was calculated, ranging from 1 to 4 [27]. Lower scores indicate better mental health status. Change in mental health was calculated by subtracting the CONOR-MHI score at baseline from the CONOR-MHI score at follow-up.

### Exposures

For primary healthcare services, we counted contact with GPs and other primary healthcare services separately. For GPs, we used the date the participant had contact with a GP, and for other healthcare services, we counted services by using the date on which a service was started, for example, the date on which in-home nursing was started. For specialist healthcare services, we used the date on which the participant had contact with a public or private hospital service, either for outpatient consultation, hospitalisation, or day treatment. Based on this, we counted the number of (1) primary healthcare services (contact with GPs and other healthcare services separately) and (2) specialist healthcare services during the pre-pandemic period (12.03.19–11.03.20) and the pandemic period (12.03.20–11.03.21) for each participant. The number of services during the pre-pandemic period was then subtracted from the number of services during the pandemic period for each service type, separately. Both primary and specialist healthcare services were then dichotomised into “no change or increased service use” and “decreased service use”.

### Analysis

All analyses were performed using Stata, version 18.0 [28]. Propensity score matching (PSM), a quasi-experimental statistical method accounting for baseline confounders from HUNT4 70 +, was applied to estimate the effect of change in health service use on each of the outcome measures [29]. The method estimates the probability (propensity score) of receiving a treatment based on background variables and matches participants with similar probabilities across treatment and control groups. This allows for the comparison of groups that are as similar as possible, enabling a more reliable estimation of the treatment effect. In this study, reductions in primary and specialist healthcare service use during the pandemic period served as the “treatment” and were our primary exposures. Age, sex, education, cohabitation status, and comorbidity served as background confounders and were the basis for the matching. The matching procedure was conducted separately for each outcome and run separately for primary healthcare services: contact with GPs and other healthcare services, and specialist healthcare services, respectively. First, logistic regression was used to estimate propensity scores, and the regression model was set up with MoCA-change, SPPB-change or CONOR-MHI-change as outcome and age, sex, education, cohabitation status, and comorbidity as predictors. Default radius matching was performed using a caliper width of 0.2 standard deviations of the logit of the propensity score [30]. Second, individuals were matched based on their propensity scores to ensure comparability between groups. Third, the average treatment effect on the treated was estimated, representing the change in outcome measures between those who experienced reduction in healthcare services and those who did not. To assess the precision of these estimates, 95% confidence intervals (95% CI) were calculated using bootstrapping with 200 repetitions. All analyses were performed in (1) the entire study sample, and (2) in women and men, separately, as cognitive-, mental-, and physical trajectories may be different in regard to sex and health care use [15–17, 20]. Group differences in continuous variables were assessed with *t*-tests, while categorical variables were evaluated using Chi-square tests. A significance level of 5% was applied. Statistics for the PSM-analysis are reported in supplementary materials, Tables S1 to S3. To assess the robustness of our findings, we conducted a sensitivity analysis using follow-up scores as outcome, while including baseline scores as confounders, Tables S4 to S6.

## Results

The included study sample (*n* = 5387) was younger than those excluded (*n* = 321, 79 years vs. 83 years, *p* < 0.001) and had a higher proportion of individuals with higher education (50% vs. 35%, *p* < 0.001). Additionally, the included study sample had higher baseline scores for cognitive function (23.8 vs. 20.3, *p* < 0.001) and physical function (9.9 vs. 7.4, *p* < 0.001), as well as lower mental health distress scores (1.5 vs. 1.6, *p* < 0.001) compared to those excluded.

Of the 5387 participants (54% women), 1983 (37%) had a reduction in contact with general practitioners (GPs) during the pandemic, while 3404 (63%) had no change or an increase in use. Among women, 1093 (38%) had reduced GP contact, compared to 890 (36%) men. For other primary care services, 315 (6%) had a reduction in use, while 5072 (94%) had no change or increased use. A reduction in other primary care service use was observed in 227 of the women (8%) and 88 (4%) of the men. Regarding specialist healthcare services, 2225 participants (41%) had a reduction in use, while 3162 (59%) had no change or an increase. Among women, 1208 (42%) used less specialist healthcare, compared to 1017 (41%) men (Table 1). Among the 2225 participants who had a reduction in specialist healthcare services, 1017 (46%) also reduced their GP contacts, while 1208 (54%) had either no change or increased GP contacts. In terms of other primary healthcare services, 196 participants (9%) showed a decrease in use, whereas 2029 (91%) had either no change or an increase in specialist healthcare services. The average changes in both primary and specialist healthcare service use are presented in Table 1.

**Table 1:** Study sample characteristics, across service use status during the pandemic

		Primary healthcare service use				Specialist healthcare service use	
		General practitioner		Other primary services			
	Total sample *N* = 5387	Decreased *n* = 1983 (37%)	No change or increased *n* = 3404 (63%)	Decreased *n* = 315 (6%)	No change or increased *n* = 5072 (94%)	Decreased *n* = 2225 (41%)	No change or increased *n* = 3162 (59%)
Sex (***n*** = 5387), ***n*** (%)							
- Women	2904 (53.9)	1093 (55.1)	1811 (53.2)	227 (78.1)	2677 (52.8)	1208 (54.3)	1696 (53.6)
- Men	2483 (46.1)	890 (44.9)	1593 (46.8)	88 (27.9)	2395 (47.2)	1017 (45.7)	1466 (46.4)
Age, mean (SD)	76 (4.7)	76 (4.7)	76 (4.7)	81 (5.6)	76 (4.5)	76 (4.7)	76 (4.7)
Education (*n* = 5381), *n* (%)							
- Primary	1317 (24.5)	486 (24.6)	831 (24.4)	134 (43.0)	1183 (23.3)	569 (25.6)	748 (23.7)
- Secondary	1302 (24.2)	475 (24.0)	827 (24.3)	89 (25.6)	1222 (24.1)	528 (23.8)	774 (24.5)
- Tertiary	2762 (51.3)	1019 (51.5)	1743 (51.3)	98 (31.4)	2664 (52.6)	1124 (50.6)	1638 (51.8)
Cohabitation (*n* = 5324), *n* (%)							
- Living alone	1540 (28.9)	570 (29.2)	970 (28.8)	163 (55.1)	1377 (27.4)	643 (29.3)	897 (28.7)
- Living with someone	3784 (71.1)	1385 (70.8)	2399 (71.2)	133 (44.9)	3651 (72.6)	1552 (70.7)	2232 (71.3)
Comorbidity (*n* = 4.972), *n* (%)							
- 0–1 self-reported disease	3126 (62.9)	1135 (62.6)	1991 (63.0)	140 (52.4)	2986 (63.5)	1228 (60.0)	1898 (64.9)
- 2 + self-reported diseases	1846 (37.1)	677 (37.4)	1169 (37.0)	127 (47.6)	1719 (36.5)	819 (40.0)	1027 (35.1)
MoCA^1^ at baseline (***n*** = 5384), mean (SD)	23.9 (3.6)	23.9 (3.6)	23.9 (3.6)	21.9 (4.4)*	24.0 (3.5)	23.7 (3.7)*	24.0 (3.5)
MoCA^1^ at follow-up (***n*** = 5305), mean (SD)	22.7 (4.4)**	22.4 (4.4)	22.7 (4.4)	19.3 (5.8)*	22.9 (4.2)	22.5 (4.5)*	22.8 (4.3)
SPPB^2^ at baseline (***n*** = 5330), mean (SD	10.3 (2.2)	10.3 (2.3)	10.3 (2.2)	8.0 (3.3)*	10.5 (2.1)	10.2 (2.3)*	10.4 (2.2)
SPPB^2^ at follow-up (***n*** = 5325), mean (SD)	9.8 (2.9)**	9.8 (3.0)	9.8 (2.9)	6.7 (3.9)*	9.9 (2.7)	9.7 (3.0)	9.8 (2.9)
CONOR-MHI^3^ at baseline (***n*** = 4774), mean (SD)	1.4 (0.4)	1.4 (0.4)	1.4 (0.4)	1.6 (0.5)*	1.4 (0.4)	1.4 (0.4)	1.4 (0.4)
CONOR-MHI^3^ at follow-up (***n*** = 4429), mean (SD)	1.5 (0.4)**	1.5 (0.4)	1.5 (0.4)	1.7 (0.5)*	1.5 (0.4)	1.5 (0.4)	1.5 (0.4)
Change in GP contacts, mean (SD)	1.7 (7.4)	− 4.8 (4.6)	5.4 (6.0)	− 1.4 (11.3)	1.9 (7.1)	0.2 (7.5)	2.7 (7.2)
Change in other primary services, mean (SD)	− 0.002 (1.5)	− 0.2 (1.5)	0.1 (1.5)	− 3.4 (3.1)	0.2 (1.0)	− 0.2 (1.6)	0.1 (1.4)
Change in specialist services, mean (SD)	− 0.1 (6.1)	− 1.1 (5.8)	0.6 (6.2)	− 2.7 (6.9)	0.1 (6.0)	− 3.8 (4.9)	2.6 (5.4)

*Significant difference between those who experienced reduced healthcare services and those who did not (*p* ≤ 0.05)

**Significant difference between baseline and follow-up on the outcome measures (*p* < 0.001)

^1^MoCA, Montreal Cognitive Assessment score, ranges 0–30 points. Higher scores indicate better cognitive function

^2^SPPB, Short Physical Performance Battery, range 0–12 points. Higher scores indicate better physical function

^3^CONOR-MHI, Cohort of Norway Mental Health Index, range 1–4 points. Lower scores indicate better mental health status

In the overall study sample, there was a decrease in cognitive and physical function and mental health scores from baseline to follow-up (Table 1). Furthermore, participants who had a reduction in other primary healthcare services had lower cognitive function, physical function, and mental health scores at both baseline and follow-up compared to those with stable or increased services. Similarly, those using less specialist healthcare services had lower cognitive function at both assessment points and lower physical function at baseline. Table 1 presents the characteristics of the study sample and the differences between those who experienced a reduction in healthcare services and those who did not.

**Fig. 1:**
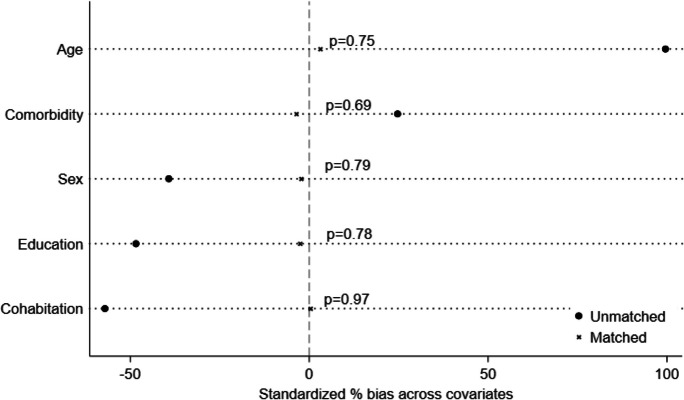
Propensity score matching for change in cognitive function measured on the Montreal Cognitive Assessment (MoCA, change from baseline to follow-up) on reduction in primary health care services. The figure illustrates the standardized % bias across covariates before (mean bias 53.5%) and after matching (mean bias 2.4%). The difference between the treated and control groups for all confounders was reduced from *p* < 0.001 for the unmatched to *p* = 0.995 for the matched. This propensity score matching example is representative of all outcome variables for both primary and specialist healthcare services

The reduction in bias due to the PSM procedure is depicted for cognitive function in Fig. 1. Before matching, there were significant differences between the treated and control groups for all confounders (*p* < 0.05). After matching, these differences were substantially reduced, indicating a successful balance across all covariates (e.g., *p* > 0.69, Fig. 1). Detailed numerical results are available in the supplementary tables, which provide full estimates.

### Primary healthcare services -contact with GPs

#### Cognitive function

Across all participants and among men, there were no significant differences in change in cognitive function scores between those who experienced a reduction in contact with GPs during the pandemic and those who did not (Table S1). Among women, a reduction in GP contact was associated with a greater cognitive decline (MoCA-change − 0.32, 95% CI-0.62, − 0.32), compared to women who had no change or an increase in such service use.

#### Physical function and mental health

No significant differences in changes in physical function or mental health scores were found between participants who experienced a reduction in GP contacts during the pandemic and those who did not (Table S1).

### Other primary healthcare services

#### Cognitive function

Across all participants, a reduction in primary care services during the pandemic was associated with a greater decline in cognitive function (MoCA-change − 0.94, 95% CI − 1.53, − 0.36), compared to those who experienced no change or an increase in such service use. Among men, a reduction in primary care services was associated with a greater cognitive decline (MoCA-change − 2.12, 95% CI − 3.13, − 1.12) compared to men who had no change or an increase in such service use. There were no significant associations between reduction in primary care services and change in cognitive function in women (Table S2).

#### Physical function

Across all participants and among women, there were no significant differences in change in physical function scores between those who experienced a reduction in primary care services during the pandemic and those who did not (Table S2). Among men, a reduction in primary care services was associated with a greater decline in physical function (SPPB-change − 1.06, 95% CI − 1.79, − 0.33), compared to those who had no change or an increased use of such services.

#### Mental health

No significant differences in change in mental health scores were found between participants who experienced a reduction in primary care services during the pandemic and those who did not (Table S2).

### Specialist healthcare services

#### Cognitive function and mental health

No significant differences in changes in cognitive function or mental health scores were found between participants who experienced a reduction in specialist care services during the pandemic and those who did not (Table S3).

#### Physical function

Across all participants and among men, there were no significant differences in change in physical function scores between those who experienced a reduction in specialist healthcare services during the pandemic and those who did not (Table S3). Among women, a reduction in specialist care services was associated with an improved physical function score (SPPB-change 0.32, 95% CI 0.11, 0.53), compared to those who had no change or an increased use of specialist care services.

### Sensitivity analysis

The results from the sensitivity analysis for GP contacts were mostly consistent with the main analysis, apart from a decline in physical function among women who experienced a reduction in GP contacts (Table S4). For other primary care services, additional findings related to a reduction in services were a greater decline in cognitive function among women, reduced physical function in the overall sample, and a more severe mental health score among men (Table S5). Finally, for specialist healthcare services, the sensitivity analysis yielded results consistent with the main analysis across all outcome measures (Table S6).

## Discussion

This population-based study found that reduced contact with GPs during the pandemic was associated with a greater decline in cognitive function among women, compared to those who had no change or an increase in GP contact. However, no such association was observed in the overall sample or among men. There were no differences in physical function or mental health scores comparing individuals with reduced GP contact and those without. For the use of other primary healthcare services, a reduction in use during the pandemic was associated with reduced cognitive function, particularly among men. A reduction in the use of these services was also associated with reduced physical function among men, while no association between reduced service usage and mental health scores was observed in the overall sample. Furthermore, there were no overall associations between reduced specialist healthcare services and change in cognitive function, physical function, or mental health scores. However, women who experienced a reduction in specialist healthcare services showed an improvement in physical function, compared to women who had no change or increased use of specialist healthcare services.

Women are more likely to experience cognitive decline than men [17, 18]. This may explain why we observed a greater reduction in cognitive function among women who had a reduction in GP visits, suggesting that women, as a more vulnerable group, were more affected by the decrease in GP visits than men. Normative studies indicate that people 70 years and older typically show an annual decline of about 0.3–0.5 MoCA points [31], suggesting that the observed − 0.32 change is small in clinical terms. Importantly, this estimate reflects the additional association with reduced healthcare use after adjusting for covariates, rather than the total cognitive decline. It suggests that women who reduced their use of GP services showed roughly one extra year of cognitive decline compared to women who did not reduce their use. While this difference is unlikely to be clinically meaningful at the individual level, even small effects may indicate increased vulnerability at the population level. Because our study has only one pre-pandemic baseline and one post-pandemic follow-up, we cannot determine sex-specific pre-pandemic trajectories. Normative MoCA data from the same source population indicate small baseline advantages for women, but provide no evidence of differential rates of decline [31]. Differential coping strategies, levels of social support, and comorbidity profiles may also have contributed to the sex-specific associations, and could not be fully accounted for in our analyses.

Our findings on the consequences of a reduction in other primary healthcare services align with the experiences described by the next of kin of people with dementia during the pandemic. They reported that reduced services led to decreased activity and cognitive stimulation [9]. Earlier research has stated that older adults reporting inadequate access to care had higher odds of cognitive impairment, compared to those who had adequate access to care, but they did not find any sex differences [19]. In our study sample, participants who experienced a reduction in other primary care services had lower cognitive scores (MoCA) compared to those who did not experience such reductions. Although a decline in cognitive function over the study period was anticipated, it appears that this decline was further exacerbated by the reduction in other primary healthcare services. This may be linked to previous findings showing that people with reduced cognition experienced a greater reduction in other primary care services during the pandemic, such as in-home nursing and day care services, compared to those with better cognitive function [2, 4]. This is likely because individuals with reduced cognitive function rely more heavily on these services and are therefore more impacted when such support is reduced or suspended.

Among men, a reduction in the use of other primary healthcare services was associated with a greater decline in both cognitive and physical function. As noted, research suggests that women’s cognitive decline is more pronounced than that of men [17]. Furthermore, physical function is influenced by health indicators (such as poor self-rated health, bodily pain, and overweight) as well as chronic diseases, with older women generally experiencing more physical limitations and a faster physical decline than men [16, 32]. Based on this, one might expect that a reduction in other primary healthcare service use would have a greater negative impact on women. However, studies have found that men tend to use other primary healthcare services less frequently than women, often delaying help-seeking until their health issues become more severe [33]. One interpretation might be that men who did use these services may have had more severe health conditions, making them particularly vulnerable to disruptions in care. Additionally, men may have fewer informal support systems or alternative coping strategies compared to women [34], which could further amplify the negative impact of reduced healthcare services on their cognitive and physical functions. However, it is worth mentioning that only a small proportion of participants (6%) had a reduction in the use of other primary healthcare services during the first year of the pandemic.

Recent research has shown a minimal decline in mental health during the early phase of the pandemic, followed by evidence of subsequent recovery [35]. These findings are in line with our study, in which we found no association between reduced primary healthcare services and mental health decline. Similarly, there were no significant differences in cognitive or physical function or mental health scores linked to reduced specialist healthcare services in the overall sample. Unlike earlier studies that focused on the early months of the pandemic [1, 4], our longitudinal design (2021–2023) captures potential longer-term consequences of reduced healthcare use. The lack of associations with mental health suggests that any short-term adverse effects may not have persisted over time. Thus, it appears that reduced availability of specialist healthcare services did not negatively impact the outcome measures we investigated.

We observed a slight increase in the average number of GP contacts among participants who had reduced use of specialist healthcare services (Table 1), suggesting that GPs may have partly compensated for reduced access to specialist healthcare services. This aligns with our earlier research, which showed an overall increase in GP use during the first six months of the COVID-19 pandemic. We had previously hypothesized that this increase was primarily driven by individuals who experienced reductions in both other primary and specialist healthcare services [2]. However, our current findings suggest that the increase in GP contacts was not necessarily dependent on reductions in other healthcare services. In fact, most participants who had no change or even an increase in primary and specialist care also maintained or increased their GP use.

Women who had a reduction in specialist healthcare service use showed improved physical function compared to those with unchanged or increased use. This is somewhat unexpected given the previously mentioned evidence that women experience a faster decline in physical functioning than men [16]. One possible explanation is that these women were healthier, more physically active, or had stronger social support networks to begin with, making them less dependent on specialist healthcare. Alternatively, they may have employed more effective coping strategies, such as drawing on informal support networks [24]. This interpretation aligns with recent findings that increased social interaction is associated with better physical performance [36]. Thus, our findings may suggest that the women in our study sample had a greater focus on health and were more socially active than men, which may have contributed to better physical functioning despite the reduction in specialist healthcare services.

Finally, our findings can be considered in light of biological processes of aging. Disruptions in care pathways may delay detection and treatment of underlying conditions, thereby exacerbating vascular disease, inflammation, and neurodegeneration, all of which contribute to cognitive and physical decline in older adults. While our study was not designed to test such mechanisms directly, acknowledging these pathways provides a broader context for understanding why reduced healthcare use may accelerate vulnerability in aging populations.

## Strengths and limitations

The strength of this study lies in its use of individual longitudinal data from a large population-based survey sample, linked to unique national registry data on the use of primary and specialist healthcare services. As the baseline data (HUNT4 70 +) were collected before the pandemic, they are unaffected by recall bias regarding health characteristics prior to the pandemic. Furthermore, the use of propensity score matching increases the likelihood of balancing baseline confounders between exposed and unexposed groups, making the comparison more comparable to that of a randomized controlled trial than traditional regression analysis [29]. However, a considerable proportion of those who required healthcare services at baseline, such as in-home nursing, day care services and hospitalization, were unable to participate in the follow-up study (HUNT AiT) due to significantly worse health, nursing home admission or death. Inclusion of these individuals may have influenced the distribution between those who used fewer services and those who did not. This is a notable limitation, as the excluded participants had lower cognitive and physical function, as well as more mental health challenges, compared to those who were included. As a result, our findings are likely to underestimate the negative health consequences of reduced healthcare use, since the frailest individuals, who may have been most affected, were not captured in the follow-up. This survivorship bias limits the generalizability of our results, particularly to the oldest old and those with severe comorbidity or advanced functional decline.

The sensitivity analysis identified some additional significant health consequences of reduced healthcare services, likely because baseline adjustment generally increases statistical power by reducing outcome variance. As this may introduce collider bias if both the treatment (i.e., reduced service use) and outcome are influenced by baseline measures (e.g. cognition), we chose to report the analysis on change scores emphasizing within-person changes over time. Although we applied propensity score matching to reduce baseline confounding, residual confounding cannot be ruled out. As this is an observational study, the findings should be interpreted as associations rather than causal effects. Some of the observed associations may partly reflect selection effects, meaning that participants who reduced their healthcare use may have differed systematically in unobserved ways (e.g., health status, health behaviors, or informal support) from those who did not. Sex-stratified findings should likewise be interpreted with caution, as pre-pandemic trajectories cannot be determined from our data. A further limitation of our study is that we cannot report how often participants received in-home nursing as part of other primary care services, as we were only able to track the initiation of such services. The small proportion of participants who reported a reduction in other healthcare services limits the generalizability of our findings. This also precluded further subdivision of other primary care services (e.g., in-home nursing, day care), as the resulting groups would have been too small to allow meaningful statistical analyses. Additionally, all participants lived in the central region of Norway near Trondheim, a Western urban hub. This may limit the generalizability of our findings to more rural areas as well as large cities in Western countries as well as non-western countries with usually less access to primary and/or specialist healthcare services. The study sample consisted primarily of individuals of Norwegian ethnicity, further restricting the external applicability of the results to other ethnic groups [21].

## Conclusion

The overall association between reduced healthcare service use during the pandemic and changes in cognitive and physical function, and mental health was limited. Most health challenges reflected as greater declines in cognitive or physical function were found to be associated with the reduction in primary care services. These findings are crucial in the context of a pandemic or other external shocks, highlighting the importance of identifying individuals with reduced abilities and ensuring they receive continued access to primary care. Notably, no negative health consequences were observed among those who had a reduction in specialist healthcare services. Women even showed improved physical function despite a decline in specialist healthcare services, although selection effects cannot be ruled out. Our findings suggest that older adults have shown adaptability throughout the pandemic despite reductions in primary and specialist healthcare service use. While a modest increase in GP contacts was noted among those who had a reduction in specialist healthcare services, the overall pattern indicates that reductions in one area of healthcare services were not offset by increases in others. Finally, the containment measures implemented in Norway, which resulted in only limited disruptions to healthcare services, appear to have been effective in mitigating adverse health outcomes.

## Supplementary Information

Below is the link to the electronic supplementary material.

Supplementary file1 (DOCX 36 KB)

## Data Availability

The data used in this study are derived from the HUNT database. Due to licensing and privacy restrictions, they are not publicly available. Access to the data can be applied for directly from the HUNT database, subject to approval.
