# Supplementary material for: Cognitive function, physical function, and mental health in older adults amid reduced primary and specialist healthcare service use during COVID-19: the HUNT study
Source: GeroScience. 2025 Sep 27;48(3):4313–25. doi: 10.1007/s11357-025-01909-x (PMC13356139; doi:10.1007/s11357-025-01909-x)
Supplement: Supplementary file 1 — Supplementary file1 (DOCX 36 KB) [file 11357_2025_1909_MOESM1_ESM.docx]

**Supplementary tables**

**Main analysis**

**Primary healthcare services**

**Table S1**. Average treatment effect* of reduced contact with general practitioners (ATT) on change in cognitive and physical function, and mental health from baseline to follow-up.

|  | ATT** | 95% CI |
| --- | --- | --- |
| Cognitive function¹ |  |  |
| - Main sample  - Women  - Men | -0.07  **-0.32**  0.10 | -0.28, 0.13  **-0.62, -0.32**  -0.18, 0.38 |
| Physical function² |  |  |
| - Main sample  - Women  - Men | 0.04  -0.13  0.10 | -0.09, 0.16  -0.33, 0.07  -0.08, 0.28 |
| Mental health³ |  |  |
| - Main sample  - Women  - Men | -0.00  -0.00  -0.00 | -0.03, 0.02  -0.03, 0.03  -0.03, 0.04 |

*** *The effect measure was the average treatment effects on those who experienced reduced services (ATT) compared to those who experienced no change or an increase, with 95% confidence intervals (bootstrapping with 200 repetitions) estimated using propensity score matching with baseline confounders as matching variables and change in cognitive and physical function, and mental health at follow-up.*

*** No change or increase in service use as reference category.*

*¹Cognitive function is measured with the Montreal Cognitive Assessment score (MoCA).*

*²Physical function is measured with the Short Physical Performance Battery (SPPB).*

*³Mental health is measured with the Cohort of Norway Mental Health Index (CONOR-MHI).*

**Table S2**. Average treatment effect* of reduction in other healthcare services¹ (ATT) on change in cognitive and physical function, and mental health from baseline to follow-up.

|  | ATT** | 95% CI |
| --- | --- | --- |
| Cognitive function (MoCA)¹ |  |  |
| - Main sample  - Women  - Men | **-0.94**  -0.64  **-2.12** | **-1.53, -0.36**  -1.35, 0.06  **-3.13, -1.12** |
| Physical function (SPPB) ² |  |  |
| - Main sample  - Women  - Men | -0.13  0.16  **-1.06** | -0.59, 0.33  -0.36, 0.67  **-1.79, -0.33** |
| Mental health (CONOR-MHI) ³ |  |  |
| - Main sample  - Women  - Men | -0.18  -0.08  0.07 | -0.09, 0.05  -0.17, 0.02  -0.04, 0.18 |

*** *The effect measure was the average treatment effects on those who experienced reduced services (ATT) compared to those who experienced no change or an increase, with 95% confidence intervals (bootstrapping with 200 repetitions) estimated using propensity score matching with baseline confounders as matching variables and change in cognitive and physical function, and mental health at follow-up.*

*** No change or increase in service use as reference category.*

*¹Other health care services as* *in-home nursing, practical assistance, daycare services, respite care, short-term nursing home stays, municipal housing, and nursing home admissions*

*² Cognitive function is measured with the Montreal Cognitive Assessment score (MoCA).*

*³ Physical function is measured with the Short Physical Performance Battery (SPPB).*

*⁴ Mental health is measured with the Cohort of Norway Mental Health Index (CONOR-MHI).*

**Specialist health care services**

**Table S3**. Average treatment effect* of reduced specialist healthcare services (ATT) on change in cognitive and physical function, and mental health from baseline to follow-up.

|  | ATT** | 95% CI |
| --- | --- | --- |
| Cognitive function (MoCA)¹ |  |  |
| - Main sample  - Women  - Men | 0.16  0.24  0.09 | -0.04, 0.36  -0.08, 0.56  -0.20, 0.37 |
| Physical function (SPPB) ² |  |  |
| - Main sample  - Women  - Men | 0.12  **0.32**  -0.09 | -0.02, 0.25  **0.11, 0.53**  -0.29, 0.10 |
| Mental health (CONOR-MHI) ³ |  |  |
| - Main sample  - Women  - Men | -0.01  -0.02  -0.00 | -0.03, 0.01  -0.05, 0.02  -0.04, 0.03 |

*** *The effect measure was the average treatment effects on those who experienced reduced services (ATT) compared to those who experienced no change or an increase, with 95% confidence intervals (bootstrapping with 200 repetitions) estimated using propensity score matching with baseline confounders as matching variables and change in cognitive and physical function, and mental health at follow-up.*

*** No change or increase in service use as reference category.*

*¹Cognitive function is measured with the Montreal Cognitive Assessment score (MoCA).*

*²Physical function is measured with the Short Physical Performance Battery (SPPB).*

*³Mental health is measured with the Cohort of Norway Mental Health Index (CONOR-MHI).*

**Sensitivity analysis**

**Primary healthcare services**

**Table S4**. Average treatment effect* of reduced contact with general practitioners (ATT) on change in cognitive and physical function, and mental health from baseline to follow-up.

|  | ATT** | 95% CI |
| --- | --- | --- |
| Cognitive function¹ |  |  |
| - Main sample  - Women  - Men | -0.12  **-0.33**  0.13 | -0.32, 0.07  **-0.64, -0.02**  -0.17, 0.43 |
| Physical function² |  |  |
| - Main sample  - Women  - Men | -0.00  **-0.26**  0.18 | -0.13, 0.12  **-0.46, -0.06**  -0.01, 0.36 |
| Mental health³ |  |  |
| - Main sample  - Women  - Men | 0.00  -0.01  0.01 | -0.02, 0.03  -0.04, 0.03  -0.02, 0.04 |

** The effect measure was the average treatment effects on those who experienced reduced services (ATT) compared to those who experienced no change or an increase, with 95% confidence intervals (bootstrapping with 200 repetitions) estimated using propensity score matching with baseline confounders as matching variable, including the baseline measure of the outcome variable, and cognitive and physical function, and mental health at follow-up as outcome variable.*

*** No change or increase in service use as reference category.*

*¹Cognitive function is measured with the Montreal Cognitive Assessment score (MoCA).*

*²Physical function is measured with the Short Physical Performance Battery (SPPB).*

*³Mental health is measured with the Cohort of Norway Mental Health Index (CONOR-MHI).*

**Table S5**. Average treatment effect* of reduction in other healthcare services¹ (ATT) on change in cognitive and physical function, and mental health from baseline to follow-up.

|  | ATT** | 95% CI |
| --- | --- | --- |
| Cognitive function*²* |  |  |
| - Main sample  - Women  - Men | **-1.20**  **-0.87**  **-2.15** | **-1.81, -0.59**  **-1.53, -0.21**  **-3.09, -1.20** |
| Physical function*³* |  |  |
| - Main sample  - Women  - Men | 0.01  0.00  **-0.14** | -0.06, 0.09  -0.10, 0.10  **0.03, 0.25** |
| Mental health*⁴* |  |  |
| - Main sample  - Women  - Men | **-0.72**  -0.17  **-0.26** | **-0.16, -0.29**  -0.63, 0.29  **-0.05, -0.47** |

*** ** The effect measure was the average treatment effects on those who experienced reduced services (ATT) compared to those who experienced no change or an increase, with 95% confidence intervals (bootstrapping with 200 repetitions) estimated using propensity score matching with baseline confounders as matching variable, including the baseline measure of the outcome variable, and cognitive and physical function, and mental health at follow-up as outcome variable.*

*** No change or increase in service use as reference category.*

*¹Other health care services as* *in-home nursing, practical assistance, daycare services, respite care, short-term nursing home stays, municipal housing, and nursing home admissions*

*² Cognitive function is measured with the Montreal Cognitive Assessment score (MoCA).*

*³ Physical function is measured with the Short Physical Performance Battery (SPPB).*

*⁴ Mental health is measured with the Cohort of Norway Mental Health Index (CONOR-MHI).*

**Specialist health care services**

**Table S6**. Average treatment effect* of reduced specialist healthcare services (ATT) on change in cognitive and physical function, and mental health from baseline to follow-up.

|  | ATT** | 95% CI |
| --- | --- | --- |
| Cognitive function (MoCA)¹ |  |  |
| - Main sample  - Women  - Men | 0.08  0.02  0.09 | -0.11, 0.27  -0.28, 0.32  -0.20, 0.38 |
| Physical function (SPPB) ² |  |  |
| - Main sample  - Women  - Men | 0.07  **0.25**  -0.12 | -0.06, 0.21  **0.06, 0.44**  -0.32, 0.07 |
| Mental health (CONOR-MHI) ³ |  |  |
| - Main sample  - Women  - Men | -0.01  -0.03  0.02 | -0.03, 0.02  -0.07, 0.00  -0.01, 0.06 |

** The effect measure was the average treatment effects on those who experienced reduced services (ATT) compared to those who experienced no change or an increase, with 95% confidence intervals (bootstrapping with 200 repetitions) estimated using propensity score matching with baseline confounders as matching variable, including the baseline measure of the outcome variable, and cognitive and physical function, and mental health at follow-up as outcome variable.*

*** No change or increase in service use as reference category.*

*¹Cognitive function is measured with the Montreal Cognitive Assessment score (MoCA).*

*²Physical function is measured with the Short Physical Performance Battery (SPPB).*

*³Mental health is measured with the Cohort of Norway Mental Health Index (CONOR-MHI).*
